# Dual Inhibition of Bcl-2/Bcl-xL and XPO1 is synthetically lethal in glioblastoma model systems

**DOI:** 10.1038/s41598-018-33784-2

**Published:** 2018-10-18

**Authors:** Enyuan Shang, Yiru Zhang, Chang Shu, Chiaki Tsuge Ishida, Elena Bianchetti, Mike-Andrew Westhoff, Georg Karpel-Massler, Markus D. Siegelin

**Affiliations:** 1 Department of Pathology & Cell Biology, Columbia University Medical Center, New York, New York, USA; 2 Department of Pediatrics and Adolescent Medicine, Ulm University Medical Center, Ulm, Germany; 3 Department of Neurosurgery, Ulm University Medical Center, Ulm, Germany; 4 Department of Biological Sciences, Bronx Community College, City University of New York, Bronx, New York, USA

## Abstract

XPO1 has recently emerged as a viable treatment target for solid malignancies, including glioblastoma (GBM), the most common primary malignant brain tumor in adults. However, given that tumors become commonly resistant to single treatments, the identification of combination therapies is critical. Therefore, we tested the hypothesis that inhibition of anti-apoptotic Bcl-2 family members and XPO1 are synthetically lethal. To this purpose, two clinically validated drug compounds, the BH3-mimetic, ABT263, and the XPO1 inhibitor, Selinexor, were used in preclinical GBM model systems. Our results show that inhibition of XPO1 reduces cellular viability in glioblastoma cell cultures. Moreover, addition of ABT263 significantly enhances the efficacy of XPO1 inhibition on the reduction of cellular viability, which occurs in a synergistic manner. While selinexor inhibits the proliferation of glioblastoma cells, the combination treatment of ABT263 and selinexor results in substantial induction of cell death, which is accompanied by activation of effector- initiator caspases and cleavage of PARP. Mechanistically we find that XPO1 inhibition results in down-regulation of anti-apoptotic Mcl-1 and attenuates ABT263 driven Mcl-1 up-regulation. Consistently, siRNA mediated silencing of Mcl-1 sensitizes for ABT263 mediated cell death and partially for the combination treatment. By using a human patient-derived xenograft model of glioblastoma in mice, we demonstrate that the combination treatment of ABT263 and Selinexor reduces tumor growth significantly more than each compound alone. Collectively, these results suggest that inhibition of XPO1 and Bcl-2/Bcl-xL might be a potential strategy for the treatment of malignant glial tumors.

## Introduction

The purpose of this study is the characterization of a novel treatment strategy for glioblastoma, a primary glial brain tumor that despite significant scientific progress still has a bad prognosis. In this context, XPO1^1,2^ has been suggested as a target for glioblastoma since recently it was shown that the compound selinexor is capable of crossing the blood brain barrier and extends survival in patient-derived orthotopic glioblastoma xenograft models^3^. Moreover, XPO1 inhibition was effective against stem-like GBM cells^3^, a fraction of cells that is known to drive resistance for therapy and recurrence. The *in vitro* efficacy of selinexor (IC_50_ – values) were reported to be in the low nano-molar range, reinforcing the potential treatment applicability of this drug.

The anti-apoptotic Bcl-2 family members are viable targets for glioblastoma given the fact that they are up-regulated in these tumors^4^. This is also supported by many preclinical studies that show that Bcl-2 family members are implicated in apoptosis regulation in model systems of these tumors. Over the last decade, several inhibitors were designed that inhibit the anti-apoptotic Bcl-2 family members^5–8^, especially Bcl-2, Bcl-xL and more recently Mcl-1, such as ABT199 and ABT263^9^. Since ABT199 has reached clinical testing and received early FDA-approval in hematological malignancies^10–12^, it is considered to be the most promising molecule out of this family. The appeal of ABT199 lies in the fact that it inhibits Bcl-2 with high-affinity, while having significantly less binding to Bcl-xL. However, the major disadvantage is that solid tumors often rely either on Bcl-xL or a combination of both Bcl-2 and Bcl-xL for their survival. Therefore, the former compound ABT263 remains still a desirable drug candidate since it dually inhibits Bcl-xL and Bcl-2 and it has reached clinical testing as well. To complicate matters further, Mcl-1 is often increased in the context of Bcl-xL/Bcl-2 inhibition, necessitating to search for strategies to counteract this compensatory increase. Earlier work has suggested that XPO1 inhibition suppresses Mcl-1 levels^3^ and therefore may be a prime candidate for sensitization to Bcl-xL inhibition mediated cell death.

In this work, we have found that XPO1 inhibition down-regulates Mcl-1 protein levels and diminished ABT263 driven Mcl-1 increase. In turn, we demonstrate that the combination treatment of ABT263 and Selinexor reduces cellular viability and tumor growth synergistically *in vitro* and in a patient-derived xenograft model of glioblastoma.

## Results

### High levels of XPO1 expression in the TCGA database confer a bad prognosis in low-grade gliomas

Although XPO1 has been established as a potential drug target for malignant glial brain tumors, we still interrogated the TCGA data base for low grade gliomas to assess as to whether or not XPO1 mRNA levels have a prognostic impact on patients with low grade gliomas. We found that high levels of XPO1 predict a worse clinical outcome with respect to survival (Supplementary Figure 1B). These findings support the notion that targeting XPO1 might be beneficial for the treatment of glial brain tumors.

### XPO1 inhibition results in synergistic reduction of glioblastoma cell growth by induction of cell death with features of apoptosis

**Figure 1:**
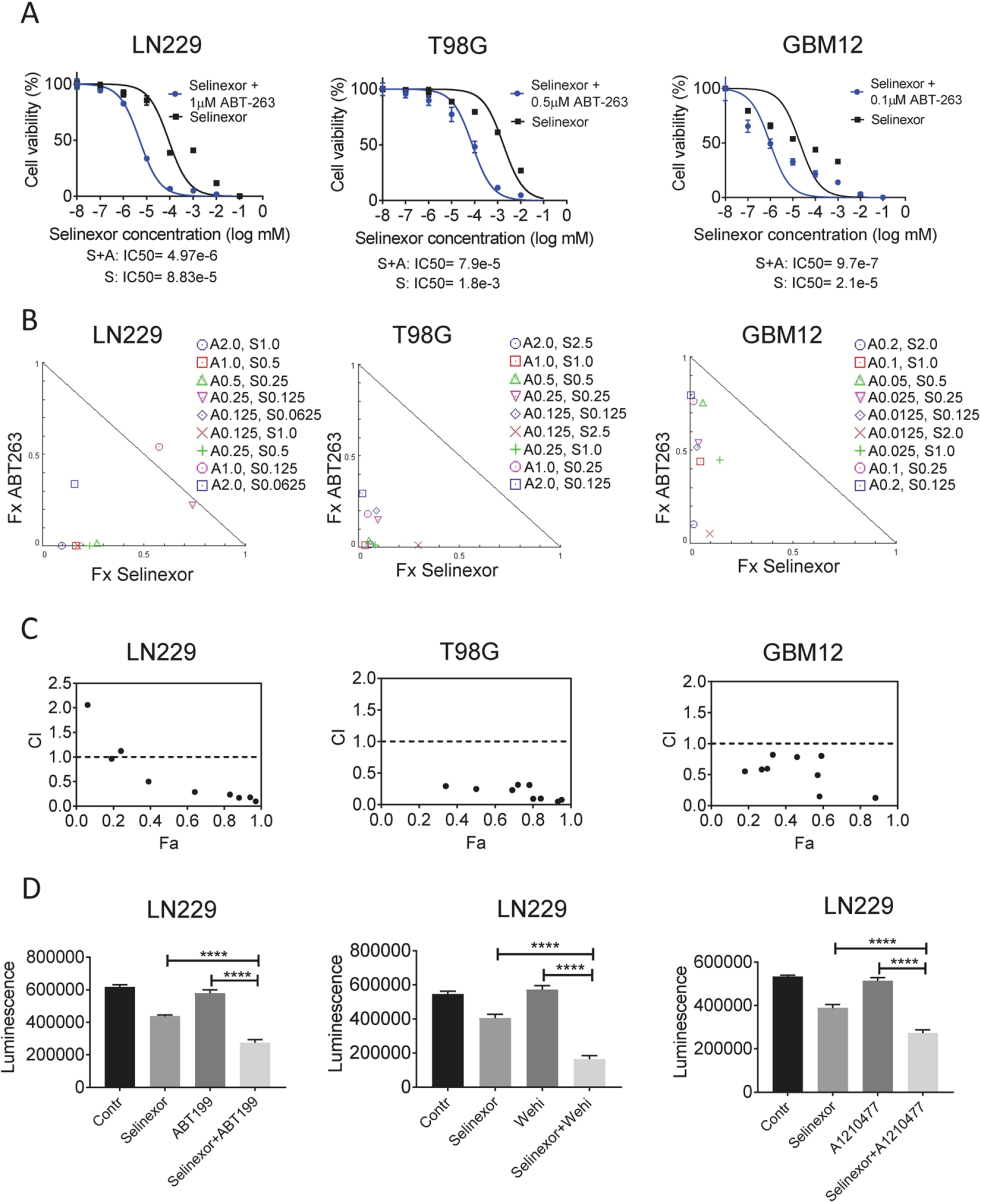
The combination treatment of BH3-mimetics and selinexor synergistically reduce cellular proliferation of glioblastoma cells. (**A**) The indicated glioblastoma cell lines, LN229, T98G and GBM12 (patient-derived xenograft) cells, were treated with increasing concentrations of selinexor in the presence or absence of the BH3-mimetic, ABT263, at 1 μM. After 72 h, cell viability assays were performed and IC_50_ values were calculated, using non-linear regression. Shown are means and SD. (**B**) LN229, T98G or GBM12 (patient-derived xenograft) cells were treated with a range of concentrations of ABT263, selinexor or the combination. 72 h after treatment, cell viability assays were performed and synergism analysis was performed. Shown are the isobolograms for the three different cell lines. Points below the border line are considered synergistic, whereas points above are antagonistic. Points on the line are additive. (**C**) LN229, T98G and GBM12 (patient-derived xenograft) cells were treated as in B. Shown is the combination index (CI) vs. the fractional response rate (Fa). CI values smaller than 1.0 indicate a synergistic interaction. (**D**) LN229 GBM cells were treated with selective BH3-mimetics, ABT199 (Bcl-2 inhibitor), WEHI-539 (Bcl-xL inhibitor) or A1210477 (Mcl-1 inhibitor) in the presence or absence of selinexor. Contr: Control, Shown are means and SD of total luminescence values. *p < 0.05; **/***/****p < 0.01.

Our findings indicate that increasing concentrations of selinexor reduce the proliferation of glioblastoma cell cultures (GBM12 (patient-derived xenograft cells, LN229 and T98G), which was most efficient in LN229 GBM cells (Fig. 1A). Since single treatment approaches are prone to fall short of expectations with regards to durability of their anti-cancer effects, we tested XPO1 inhibition in the context of a novel combination therapy, involving BH3-mimetics. Given the efficacy of Bcl-xL inhibition in solid malignancies, we initiated our studies with ABT263. Unequivocally, we found that ABT263 potently reduced the IC_50_ values of selinexor in all GBM cells tested (Fig. 1A), suggesting that selinexor and ABT263 act synergistically to reduce proliferation. Similar results were also obtained in stem-like GBM cells, NCH644 (Supplementary Figure 1C). In order to formally evaluate synergism, we tested the combination therapy of ABT263 and Selinexor over a broad range of concentrations. We found that based on an synergism analysis selinexor and ABT263 synergistically reduced the proliferation of LN229, T98G, GBM12 and U87 GBM cells (Fig. 1B,C and Supplementary Figure 1D).

Next, we determined the functional implications of anti-apoptotic Bcl-2 family members on the anti-proliferative effect of the combination therapy. To this end, we took advantage of the recently developed selective BH3-mimetics, ABT199 (Bcl-2), WEHI-539 (Bcl-xL) and A1210477 (Mcl-1). While all selective BH3-mimetics enhanced the reduction of cellular proliferation mediated by selinexor, the most efficacious compound was the Bcl-xL inhibitor, WEHI-539 (Fig. 1D and Supplementary Figure 1A).

**Figure 2:**
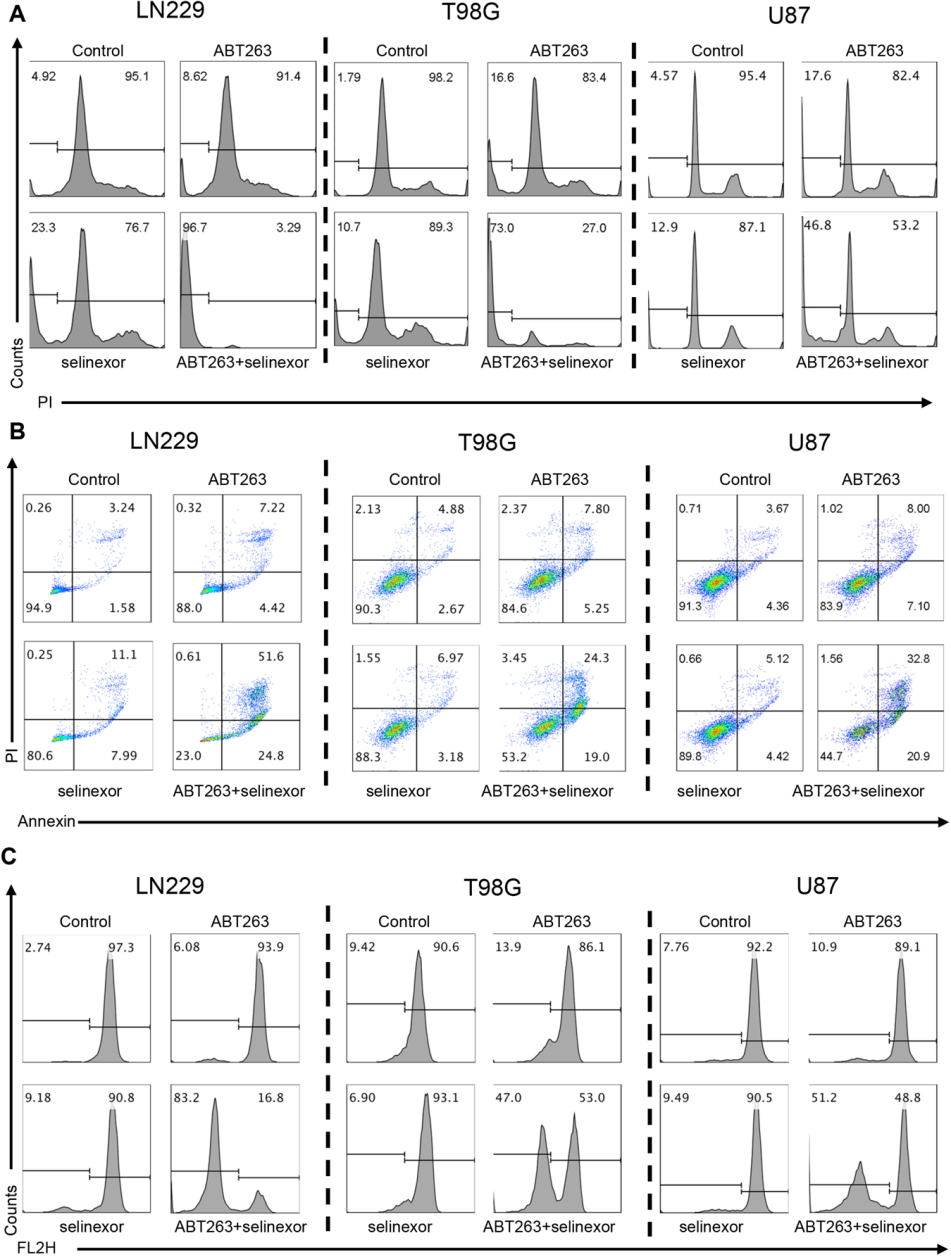
The combination treatment of ABT263 and selinexor shows features of apoptotic cell death. (**A**) LN229, T98G and U87 GBM cells were treated with ABT263, selinexor or the combination. After 72 h, cells were harvested, fixed, stained with propidium iodide and analyzed by flow cytometric analysis for DNA – fragmentation. Shown are representative flow cytometry plots. (**B**) The same set of cell lines as in A were treated with the indicated drugs and the same conditions (except for the incubation time, which was 24 h). Thereafter, cells were stained with Annexin V/Propidium iodide and analyzed by multi-parametric flow cytometry. Shown are representative plots. (**C**) The same set of cell lines as in A were treated with the indicated drugs and the same conditions (except for the incubation time, which was 24 h). Thereafter, cells were stained with TMRE and analyzed by flow cytometry. Shown are representative flow plots.

**Figure 3:**
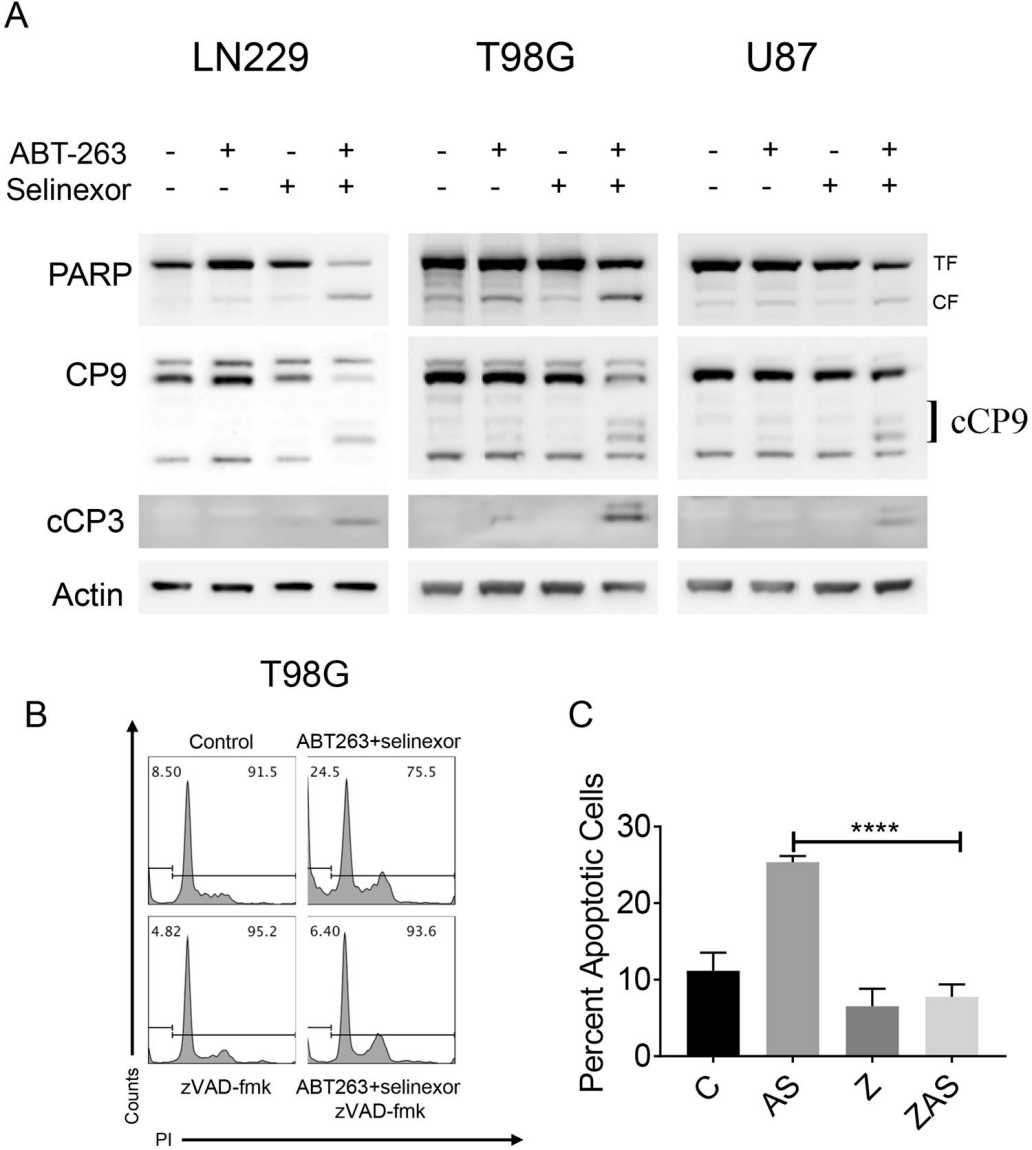
The combination treatment of ABT263 and selinexor elicits apoptosis in a partial caspase-dependent manner. (**A**) LN229, T98G and U87 GBM cells were treated with ABT263, selinexor or the combination. Thereafter, whole-cell protein lysates were harvested and analyzed by conventional western blotting for the expression of PARP, caspase-9 (CP9), cleaved caspase-3 (cCP3) and Actin. TF: total form, CF: cleaved form. (**B**) T98G cells were treated with vehicle or the combination treatment of ABT263+ selinexor in the presence or absence of pan-caspase inhibitor, zVAD-fmk. Thereafter, cells were fixed, stained with propidium iodide and analyzed by flow cytometry. Shown are representative flow plots. (**C**) The experiment in B was quantified and statistical analysis was performed. (**C**) Control, AS: ABT263+ Selinexor, Z: zVAD-fmk, ZAS: ZVAD-fmk + ABT263+ Selinexor. Shown are means and SD. *p < 0.05; **/***/****p < 0.01.

Based on the notion that essentially almost all combination therapies, involving BH3-mimetics, work through enhancement of intrinsic apoptosis, we determined activation of apoptosis induced by the combination treatment of selinexor and ABT263, utilizing several state-of-the art methods. First, we treated our three GBM cell lines with ABT263, selinexor and the combination treatment of ABT263+ Selinexor and subsequently performed DNA-staining, followed by flow-cytometric analysis. These experiments undoubtedly showed that the combination treatment induces significantly more DNA-fragmentation than vehicle or single treatments (Fig. 2A and Supplementary Figure 2). Second, to more specifically measure apoptosis, we conducted Annexin V/propidium iodide staining. Akin to the DNA-fragmentation, we found the highest proportion of Annexin V positive cells in the combination treatment (Fig. 2B). Third, we measured mitochondrial membrane potential after treatment with vehicle, ABT263, selinexor and the combination treatment since loss of mitochondrial membrane potential precedes release of cytochrome-c from mitochondria, that in turn activates the apoptosome with cleavage of caspase-9. As anticipated, the combination treatment more potently disrupted mitochondrial membrane potential than vehicle or single treatment in all three cell lines (Fig. 2C). Finally, we determined activation of initiator- (caspase-9) and effector-caspases (caspase-3) in the context of our various treatments by performing western blot analysis of the total as well as the cleaved forms of caspases. It is well accepted that cleavage of caspases correlates with their activation. We found that the combination treatment led to a profound cleavage of caspases with reduction/disappearance of the total (inactive) forms (Fig. 3A). Akin to caspases, the down-stream substrate of caspases PARP was cleaved as well, providing further evidence for the activation of the caspase cascade (Fig. 3A). It should be noted that suboptimal dosages of ABT263 were used and therefore single treatment with ABT263 does not elicit caspase cleavage.

Activation of caspases on its own may not have functional implications. To this purpose, we utilized the pan-caspase inhibitor, zVAD-fmk. We found that zVAD-fmk partially rescued from apoptosis induced by the combination treatment, indicating at least a partial implication of caspases in the death (Fig. 3B,C). Collectively, these findings suggest that the combination treatment elicits its anti-glioma effects in part through cell death with features of apoptosis.

### XPO1 inhibition modulates the levels of anti-apoptotic Bcl-2 family members

**Figure 4:**
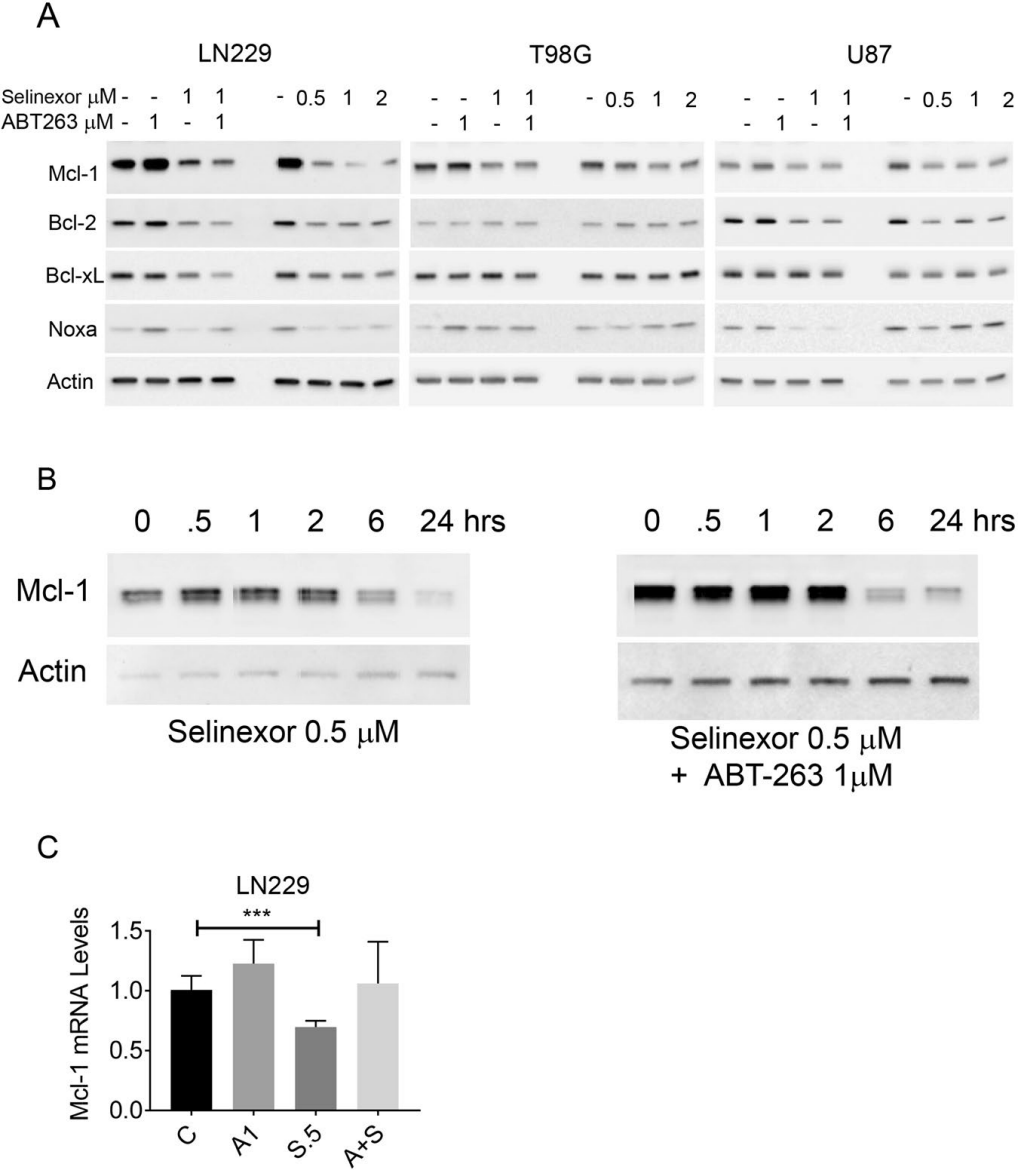
Inhibition of XPO1 by selinexor regulates the expression of anti- and pro-apoptotic Bcl-2 family member proteins. (**A**) LN229, T98G and U87 GBM cells were treated with ABT263, selinexor or the combination. Alternatively, cells were treated with increasing concentrations of selinexor for 24 h. Thereafter, whole cell protein lysates were collected and analyzed by conventional western blotting for the expression of Mcl-1, Bcl-2, Bcl-xL, Noxa and Actin. All concentrations are in μM. (**B**) LN229 GBM cells were treated with selinexor or the combination treatment of ABT263+ selinexor for the indicated time points (time course experiment). Thereafter, whole cell protein lysates were prepared and analyzed for the expression of Mcl-1. Hrs: hours. (**C**) LN229 GBM cells were treated with vehicle, ABT263, selinexor or the combination treatment. After 6 h, RNA was harvested, reverse transcribed and subjected to real-time PCR analysis for Mcl-1.

Key regulators for intrinsic apoptosis are the pro- and anti-apoptotic Bcl-2 family molecule and it is well accepted that Mcl-1 mediates resistance towards BH3-mimetics that target Bcl-2 and Bcl-xL, but not Mcl-1. For this reason, we analyzed the expression of Bcl-2 family members upon treatment with selinexor in three cell lines (Fig. 4A). While Bcl-xL was unchanged, selinexor suppressed Mcl-1 protein levels. Bcl-2 was down-regulated in LN229 and U87, respectively, but not in T98G. We also evaluated the expression of pro-apoptotic Noxa, an intrinsic antagonist of Mcl-1. While in LN229 we noted a decrease in Noxa levels, we found an increase in T98G and U87 cells upon selinexor administration (Fig. 4A). The suppression of Noxa in LN229 is likely a result of Mcl-1 suppression since Mcl-1 and Noxa interact with each other and Mcl-1 suppression was the most strongest in LN229 cells upon selinexor treatment (Fig. 4A). However, the ratio of Noxa and Mcl-1 is shifted towards a pro-apoptotic state.

Given the known impact of ABT263 on the levels of Bcl-2 family members, we evaluated the expression of Bcl-2, Bcl-xL and Mcl-1 upon ABT263, Selinexor and Selinexor +ABT263 treatment. While ABT263 up-regulated Mcl-1 protein levels, this increase was attenuated in the presence of selinexor, thus mediating a pro-apoptotic state (Fig. 4A). To better appreciate the course of the events, we conducted a time-course analysis for Mcl-1 levels upon selinexor or ABT263+ selinexor treatment. Our findings show that Mcl-1 protein levels are suppressed as early as 6 h by selinexor, demonstrating that Mcl-1 decrease is an early event (Fig. 4B).

Moreover, we determined Mcl-1 mRNA levels upon ABT263, selinexor and the combination treatment. We found that selinexor slightly reduced Mcl-1 mRNA levels, but this suppression was less as compared to the reduction on protein level (Fig. 4C). Moreover, while the combination treatment of ABT263+ Selinexor did not reduce the Mcl-1 mRNA levels, it strongly suppressed protein levels (Fig. 4B,C). Therefore, it is highly likely that selinexor affects Mcl-1 levels at the transcriptional and posttranslational level.

### Down-regulation of Mcl-1 is a central mechanism by which selinexor sensitizes glioblastoma cells for ABT263 mediated cell death with features of apoptosis

**Figure 5:**
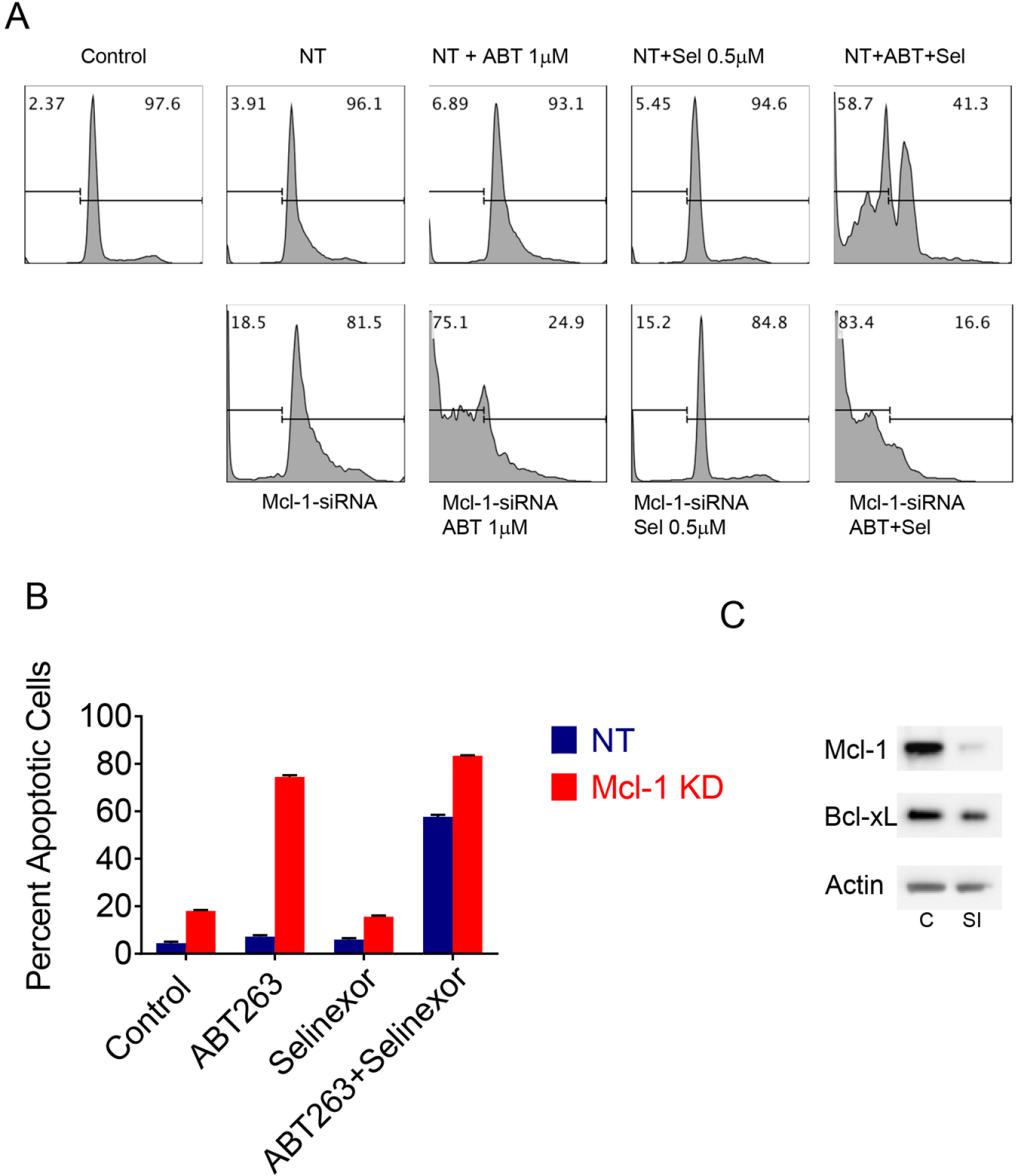
Mcl-1 is the key regulator in the combination treatment, involving ABT263 and Selinexor. (**A**) LN229 GBM cells were transfected with non-targeting or Mcl1-specific siRNA for 72 h. Non-targeting and Mcl-1 specific siRNA transfected cells were subsequently exposed to vehicle, ABT263 (ABT), selinexor (Sel) or the combination treatment of ABT263 and selinexor (ABT + Sel). Thereafter, cells were fixed, labeled with propidium iodide and analyzed by flow cytometry. Shown are representative plots. (**C**) Control; SI: Mcl-1 specific siRNA.; NT: non-targeting siRNA. (**B**) Shown are the quantifications obtained from experiment performed in A. Shown are means and SD. (**C**) LN229 GBM cells were transfected as in A. Whole cell protein lysates were collected and analyzed for the expression of Mcl-1 and Bcl-xL by standard western blotting (C: Control, non-targeting siRNA; SI: siRNA Mcl-1).

Since Mcl-1 levels were predominantly affected by selinexor treatment, we proceeded to test the hypothesis that Mcl-1 is likely a functional mediator in cell death mediated by the combination treatment. To this purpose, we used Mcl-1 specific siRNA and silenced the expression of Mcl-1 in LN229 GBM cells (Fig. 5C). Silencing was validated by western blotting. After transfection, LN229 transfected with non-targeting or Mcl-1 specific siRNA were subjected to treatment with vehicle, ABT263, Selinexor and ABT263+ Selinexor. While non-targeting siRNA had minimal effects on DNA-fragmentation, Mcl-1 silencing led to a mild to moderate increase in DNA-fragmentation, suggesting that Mcl-1 is important for survival of LN229 cells (Fig. 5A,B). We found that Mcl-1 silencing drastically sensitized for ABT263 mediated cell death, whereas selinexor mediated DNA-fragmentation was not enhanced as compared to the Mcl-1 siRNA alone (Fig. 5A,B). Finally, we evaluated the effect of Mcl-1 silencing on the combination treatment of ABT263+ selinexor. Mcl-1 silencing further enhanced the effects of the combination treatment on cell death (Fig. 5A,B). A second experiment was performed in T98G cells, which mirrors the findings obtained in LN229 cells (Supplementary Figure 3A–C). Taken together, these observations strongly suggest that Mcl-1 is a key mediator of cell death mediated by the combination treatment.

### The combination treatment of ABT263 and Selinexor reduces tumor growth in a murine patient-derived xenograft model of glioblastoma

**Figure 6:**
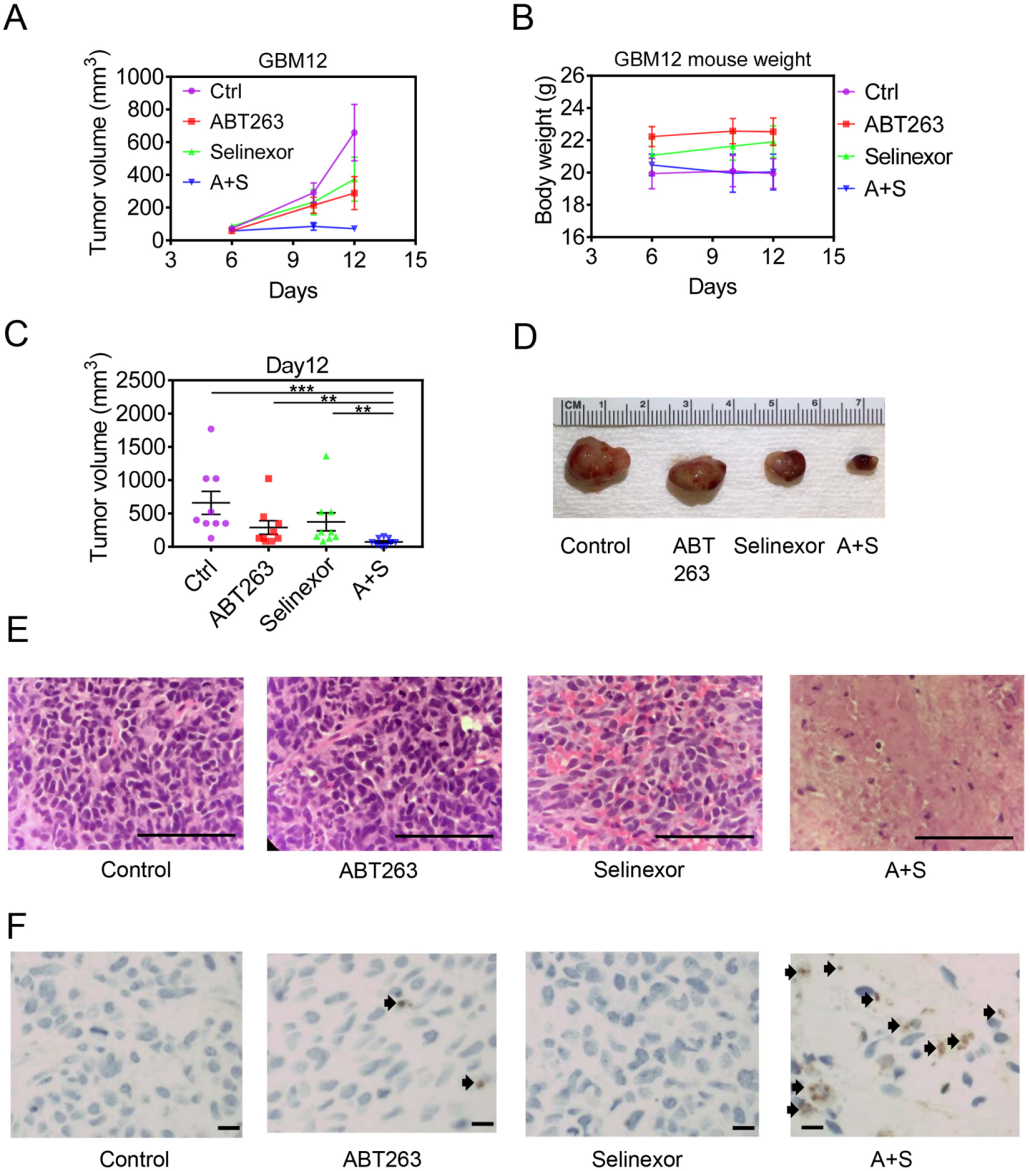
The combination treatment of ABT263 and selinexor reduces tumor growth stronger than vehicle or single treatments in a patient-derived xenograft model of human GBM. (**A**) Patient-derived xenograft model (GBM12) was implanted subcutaneously in nude mice. Once tumors became palpable and were about to enter an exponential growth rate, four treatment groups were randomly assigned: A, Vehicle (Ctrl), ABT263, selinexor or ABT263 + selinexor (A + S). Two treatments were given, consisting of vehicle, 75 mg/kg ABT263, 10 mg/kg selinexor or the combination. Tumor growth was measured and is plotted as volume over time (days). Shown are means and SD. (**B**) for the same experiments described in A weight measurements were taken for the individual groups. (**C**) On the day of conclusion of the experiment described in A, statistical analysis was performed. Shown are means and SD. (**D**) Representative gross images of the individual groups are shown after conclusion of the experiment. A + S: ABT263 + selinexor. (**E**) Tumors from the individual groups were fixed, embedded in paraffin and paraffin section were stained with standard hematoxylin and eosin staining (H&E). Shown are representative images from the individual treatment groups. F, Tumors from the indicated groups were stained with TUNEL. Representative images are shown and arrows highlight TUNEL positive cells. *p < 0.05; **/***/****p < 0.01.

The assessment as to whether or not a proposed preclinical drug combination is efficacious in animal models is of highest importance since it allows to determine *in vivo* efficacy while at the same time it provides information about potential toxicities. The emergence of patient-derived xenograft has greatly assisted to ensure that *in vivo* models are biologically closest to patients‘ tumors. Therefore, we determined the *in vivo* efficacy of ABT263, Selinexor and the combination in a patient-derived xenograft model of glioblastoma. We utilized the GBM12 models in a manner similar as described in previous studies. In this regard, the subcutaneous model system provides a relevant and resource-efficient approach to assess treatment efficacy and toxicity *in vivo*. After the establishment of PDX tumors, four treatment groups were established: A, Vehicle, B, ABT263, C, Selinexor, D, ABT263+ Selinexor. Treatment was administered until the defined endpoint of the study was reached. While both selinexor and ABT263 showed some suppression of tumor growth, the combination treatment was most potent, confirming our *in vitro* findings (Fig. 6A–E). Despite this significant efficacy, it is noteworthy that there was no toxicity associated with the combination treatment as determined by weight measurement and general health assessment of the animals during and after the treatments (Fig. 6B). We further asked the questions by which manner ABT263+ Selinexor exerted these potent growth inhibitory effects. Based on the *in vitro* findings, it was highly likely that this involves induction of cell death. However, given the quite different settings we validated this hypothesis by staining tumor tissue with standard HE staining from each individual treatment group. As anticipated, the control tumors displayed a high cell density with numerous mitotic figures with few areas of necrosis (Fig. 6E). In contrast, the combination treatment demonstrated vast areas of tumor cell necrosis accompanied by TUNEL positive cells, supporting the notion that the combination treatment exerted its biological effects predominantly through cell death induction (Fig. 6E,F). This notion is of significance since a tumor regression may only be accomplished by treatments that have the ability to induce cell death.

## Discussion

The quest for more durable treatment approaches remains a priority in cancer research given the fact that many malignancies still harbor a dismal prognosis^13^. Belonging to this group are malignant glial tumors, such as glioblastoma^14^. Recent changes in the classification scheme of these tumors have been made, which refers in particular to a discovery of mutations in the IDH1 gene^14^. While a huge portion of novel findings with regards to the molecular classification has been made, therapeutic advances lack behind. In light of the fact that an abundance of small molecule inhibitors that specifically target newly discovered alterations it is of high relevance to determine potential therapeutic treatment regimens, involving these compounds or combination of them^15–18^. As stated earlier, drug combinations are likely to be the key for the identification of novel treatments since virtually all tumors depend on multiple signaling cascades^18–21^. In this work, we have followed this strategic pattern and describe a novel synthetic lethal interaction between XPO1 and Bcl-2/Bcl-xL inhibition. To accomplish this, we took advantage of the XPO1 inhibitor, selinexor, and the Bcl-2/Bcl-xL inhibitor, ABT263^1–3^. The combination of these compounds potently induced cell death with features of apoptosis in several glioblastoma culture model systems in a highly synergistic manner. More relevantly, we tested the combination treatment in a patient-derived xenograft model of human GBM and found that tumors treated with ABT263 and Selinexor were significantly smaller than tumors treated with single compounds or vehicle. It is also notable that we did not detect any form of toxicity throughout that treatment. To the best of our knowledge, we are the first to describe such observations in the context of malignant glial brain tumors. Given the fact that selinexor penetrates the blood-brain barrier and has reached phase-II clinical testing, such a combination therapy is a potential viable option for patients. However, it should be noted that the model systems used here does not have a blood-brain barrier (due to subcutaneous tumor localization). In addition, a further limitation is that the micro-environment in an orthotopic location is different from the one in the cutis. While our observations are restricted to glioblastoma, it is noteworthy that this approach might have a role in other solid tumors as well. It is well accepted that the anti-apoptotic Bcl-2 family members play a significant role in other major solid malignancies, such as colon, lung and breast cancer, which are far more frequent than malignant glial brain tumors^22^.

Mechanistically, we demonstrated that cell death with features of apoptosis is activated by the combination treatment and that this is likely be mediated through the impact of selinexor on Mcl-1 protein levels. We cannot exclude the possibility that other forms of cell death, such as non-caspase dependent mediated cell death, are contributing to our assessed drug-synergy. With regards to Mcl-1, we validated its involvement by siRNA experiments. Mcl-1 is a bona-fide mediator of resistance towards BH3-mimetics that target either Bcl-2, Bcl-xL or the combination of the two. More recently, selective Mcl-1 inhibitors were designed and in these molecules are potentially amenable to clinical translation. Akin to the former BH-3 mimetics, ABT737 and ABT263, Mcl-1 inhibitors might be particularly well-suited for drug combination therapies^7,15^. In the context of glioblastoma, public databases suggest that Mcl-1 is up-regulated in these tumors as compared to normal brain tissue. The pitfall of these Mcl-1 specific BH3-mimetics is their relatively large molecular size, which may dampen the penetrance of this compound class through the blood brain barrier. It is also for this reason that other modalities or compounds are necessary to interfere with Mcl-1 levels in high grade gliomas. Selinexor may be such a molecule since it penetrates the blood-brain-barrier and lowers Mcl-1 levels. Other strategies to lower Mcl-1 levels are to lower its transcription, synthesis or in particular to enhance its proteasomal degradation^19,23–26^. Concerning proteasomal degradation, it is well known that Mcl-1 is an unstable protein that through phosphorylation/dephosphorylation is subjected to proteasomal degradation^19,23–26^.

Collectively, we have provided a foundation to further develop a drug combination that targets Bcl-2/Bcl-xL and XPO1.

## Materials and Methods

### Reagents

Selinexor, ABT263, ABT199, WEHI-539 and A1210477 were purchased from Sellekchem.

### Cell cultures and growth conditions

U87MG, LN229 and T98G human glioblastoma cell lines were obtained from the American Type Culture Collection (Manassas, VA). The respective cell line depository authenticated the cells. The NCH644 stem-like GBM cells (non-adherent) were purchased from (CLS, Heidelberg, Germany) and cultured in MG-43 medium (CLS, Heidelberg, Germany) for maintenance and experiments. The GBM12 cells were extracted from the tumor and cultured as indicated in detail in the following reference^27^.

### Cell viability assays

Viability assays were performed as previously described^28–30^. 4000 cells were seeded in 96-well plates prior treatments with the indicated BH3-mimetics (ABT263, WEHI-539, A1210477, ABT199) or the XPO1 inhibitor, selinexor. Briefly, anti-proliferative effects were determined by using the CellTiter-Glo (Promega, Madison, WI) luminescent cell viability assay in 96-well plates 72 h after treatment according to the protocol as described by the manufacturer. We utilized the CompuSyn software (ComboSyn, Inc., Paramus, NJ) to assess drug synergism, which involves the computation of the combination index (CI). A CI <1 was defined as synergistic, a CI = 1 as additive and a CI >1 as antagonistic.

### Measurement of apoptosis and mitochondrial membrane potential

For Annexin V/propidium iodide staining the Annexin V Apoptosis Detection Kit (BD Pharmingen) was used as previously described^31,32^. TMRE staining was performed according to the manufacturer’s instructions (Mitochondrial Membrane Potential kit, Cell Signaling Technology, Danvers, MA). The data were analyzed with the FlowJo software (version 8.7.1; Tree Star, Ashland, OR).

### Transfections of siRNAs

Transfections with non-targeting or Mcl-1 specific siRNAs were performed with Oligofectamine® 2000 (Invitrogen, Carlsbad, CA) or Oligofectamine as described in^31,32^.

### Western blot analysis

Specific protein expression in cell lines was determined by Western blot analysis as described before. Uncropped gel images are provided in the supplementary section.

### Real-time PCR analysis

Real-time PCR analysis was performed for Mcl-1 with primers and methodology as earlier described^33,34^.

### Subcutaneous patient-derived xenograft model

The GBM12 PDX model was used for *in vivo* efficacy assessment. This model was established and described by Dr. Jann Sarkaria^35^. Selinexor (10 mg/kg) and ABT263 (75 mg/kg) were dissolved in 10% DMSO, 32% Cremophor EL (SIGMA, St. Louis, MO), 8% Ethanol (Pharmco-Aaper, Brookfield,CT) and 50% PBS. Treatments were administered on day 6 and 10 (after cell implantation) at the dosage described above (intraperitoneal injections).

### Statistical analysis

Statistical significance was assessed by Student’s t-test using Prism version 7.00 (GraphPad, La Jolla, CA). A p ≤ 0.05 was considered statistically significant. Drug synergy analysis was performed to detect synergistic, additive or antagonistic effects as previously described^36,37^.

### Ethical approval

All procedures were in accordance with Animal Welfare Regulations and approved by the Institutional Animal Care and Use Committee at the Columbia University Medical Center.

## Electronic supplementary material

Supplementary Dataset 1

## Data Availability

All data generated or analyzed during this study are included in this published article (and its Supplementary Information files).
