# Supplementary material for: Dual Inhibition of Bcl-2/Bcl-xL and XPO1 is synthetically lethal in glioblastoma model systems
Source: Sci Rep. 2018 Oct 18;8:15383. doi: 10.1038/s41598-018-33784-2 (PMC6194027; doi:10.1038/s41598-018-33784-2)

Dual Inhibition of Bcl-2/Bcl-xL and XPO1 is synthetically lethal in glioblastoma model systems

Enyuan Shang, Yiru Zhang, Chang Shu, Chiaki Tsuge Ishida, Elena Bianchetti, Mike-Andrew Westhoff, Georg Karpel-Massler and Markus D. Siegelin

Supplementary figure legends:

Supplementary Figure 1: XPO1 is a prognostic marker in low-grade gliomas

A, LN229 GBM cells were treated with selinexor, ABT263 or the combination for 72h. Thereafter, cellular viability assay was conducted as described in the main figures. Shown are means and SD of luminescence units. B, The TCGA database for low-grade gliomas (all tumors form the database were considered) was interrogated for prognostic information with regards to mRNA level expression of XPO1. Compared are high (95th-100th percentile) and low-level expressing tumors (1st-5th percentile) and survival is plotted as a Kaplan-Meier-curve. A p-value was calculated. C, NCH644 stem like GBM cells (CLS GmbH, Eppelheim, Germany) were treated with selinexor in the presence or absence of ABT263 and analyzed for cellular viability as in main Figure 1A. Shown are means and SD. Non-linear regression analysis was performed to calculate the IC50 values. D, U87 GBM cells were treated with different concentrations of ABT263 and selinexor. Shown is the combination index (CI) vs. the fractional response rate (Fa). CI values smaller than 1.0 indicate a synergistic interaction.

Supplementary Figure 2: XPO1 and Bcl-2/Bcl-xL inhibition leads to enhanced DNA – fragmentation in glioblastoma cells

A, B, C, LN229, T98G and U87 GBM cells were treated with vehicle (control), ABT263, selinexor and the combination treatment as indicated. Thereafter, cells were fixed, labeled with propidium iodide and read on a flow cytometer in the red channel. Shown are means and SD. These are the quantifications from main figure 2a. A: ABT263, S: selinexor, AS: ABT263+selinexor. Concentrations are in μM. **** indicates a p-value of less than <0.0001.

Supplementary Figure 3: Impact of silencing of Mcl-1 on selinexor, ABT263 or ABT263+selinexor mediated DNA - fragmentation

A, T98G cells were transfected with non-targeting (nt) or Mcl-1 specific siRNA and treated with ABT263 (ABT), selinexor (Sel) or the combination of both. Thereafter, cells were fixed, stained with PI and analyzed for DNA – fragmentation. Shown are representative flow plots. B, T98G cells were transfected with non-targeting (NT) or Mcl-1 specific siRNA (SI) and analyzed for the expression of Mcl-1 and Actin by standard western blotting. C, The results in A were quantified for the amount of apoptotic cells in each condition.

Supplementary Figure 1

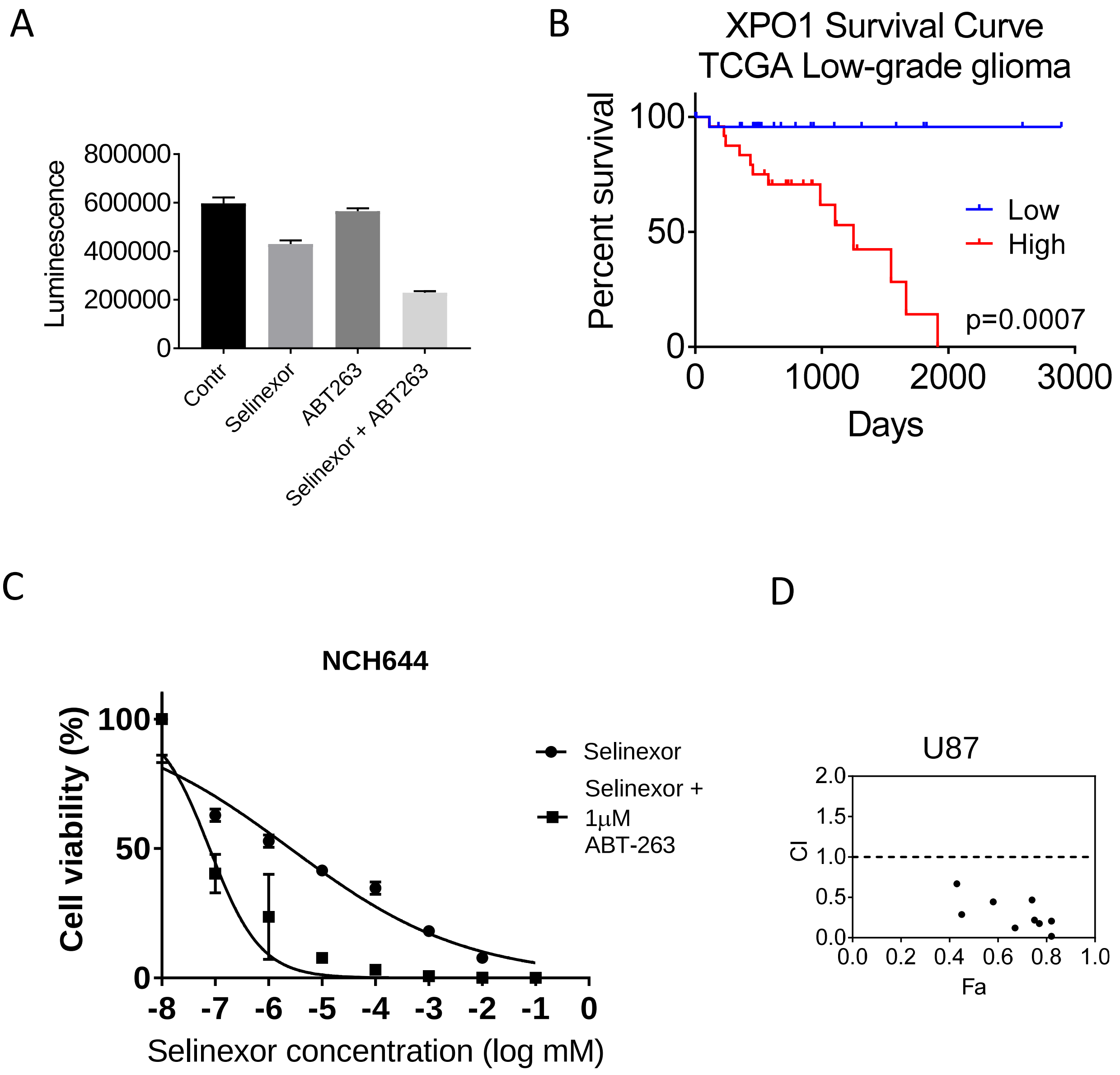

Sel IC50: 2.5e-6

Sel+ABT IC50: 7.8e-8

Supplementary Figure 2

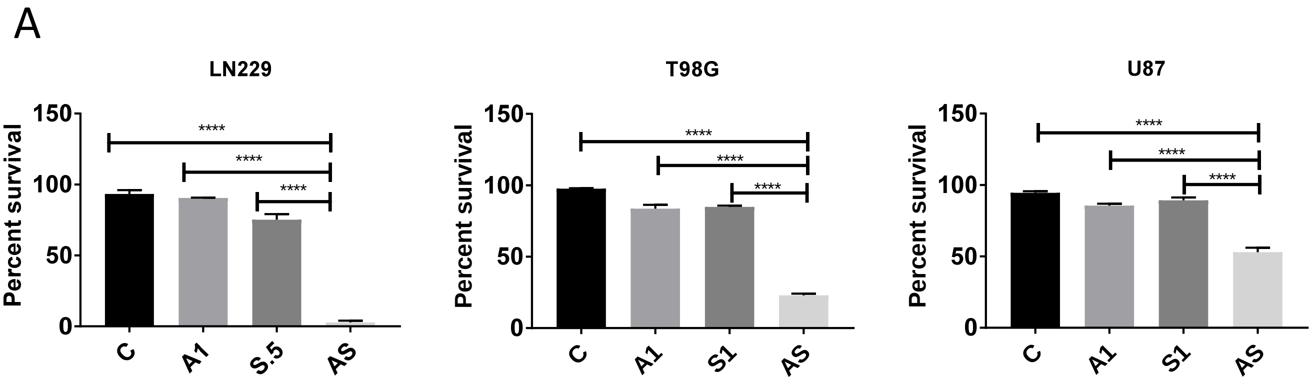

Supplementary Figure 3

A

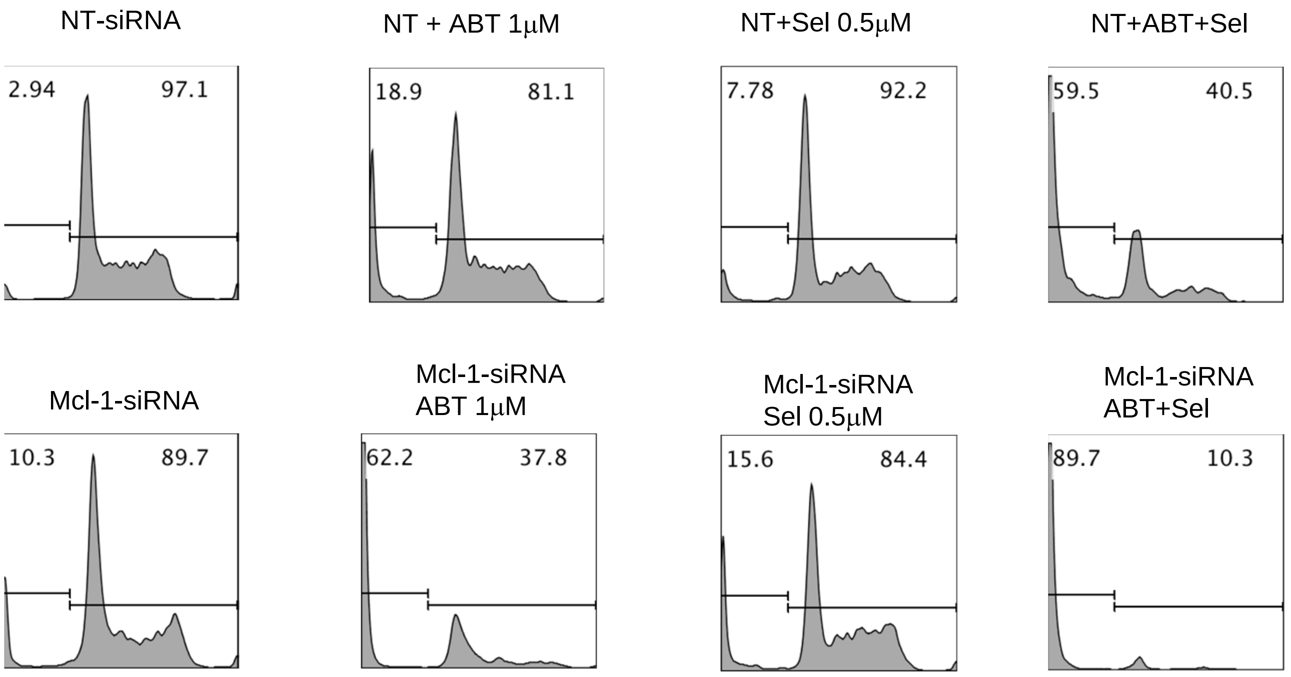

B

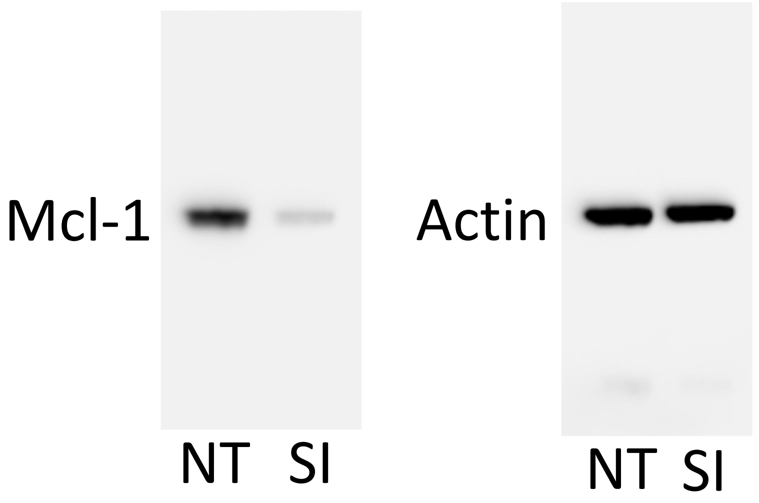

C

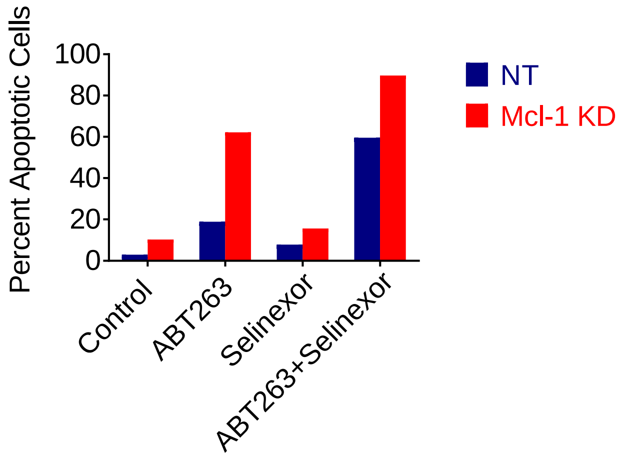

Uncropped western blot images

Figure 3S

A

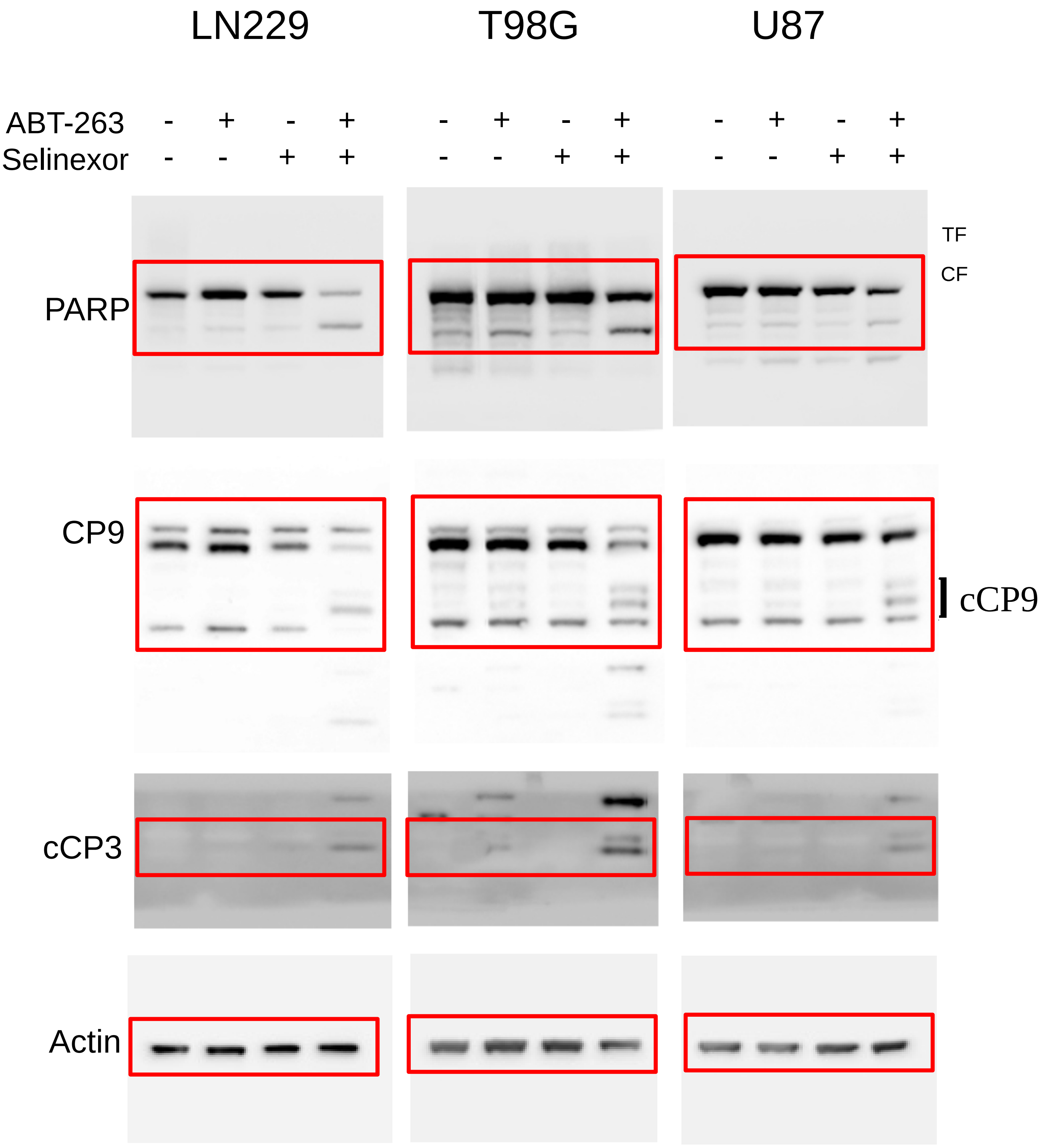
LN229
T98G
U87
ABT-263 - + - + - + - + - + - +
Selinexor - - + + - - + + - - + +
PARP
TF
CF
CP9
cCP9
cCP3
Actin

Figure 4S

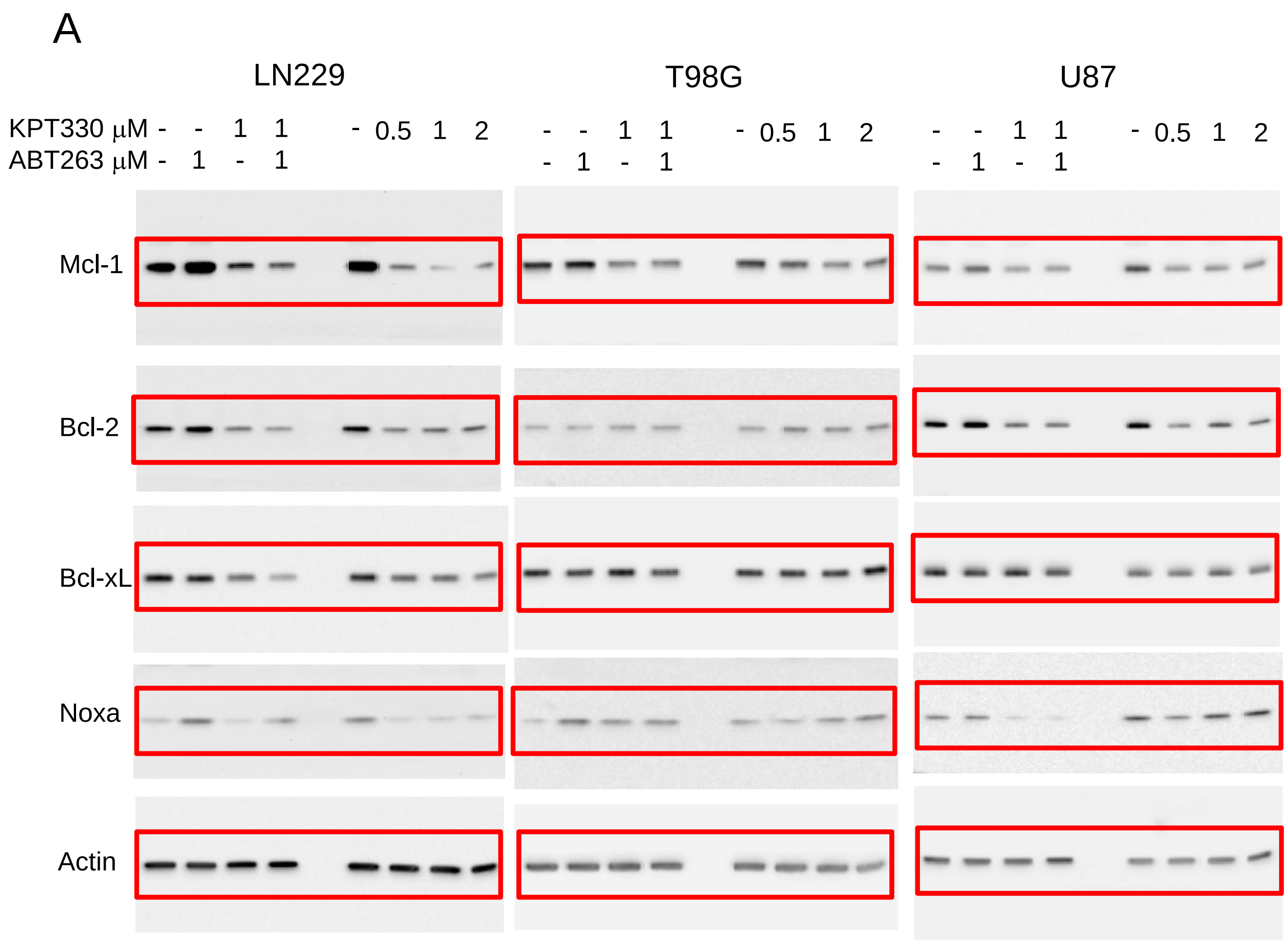
A
LN229
T98G
U87
KPT330 µM - - 1 1 - 0.5 1 2
ABT263 µM - 1 - 1
- - 1 1 - 0.5 1 2
- 1 - 1
- - 1 1 - 0.5 1 2
- 1 - 1
Mcl-1
Bcl-2
Bcl-xL
Noxa
Actin

Figure 4S

B

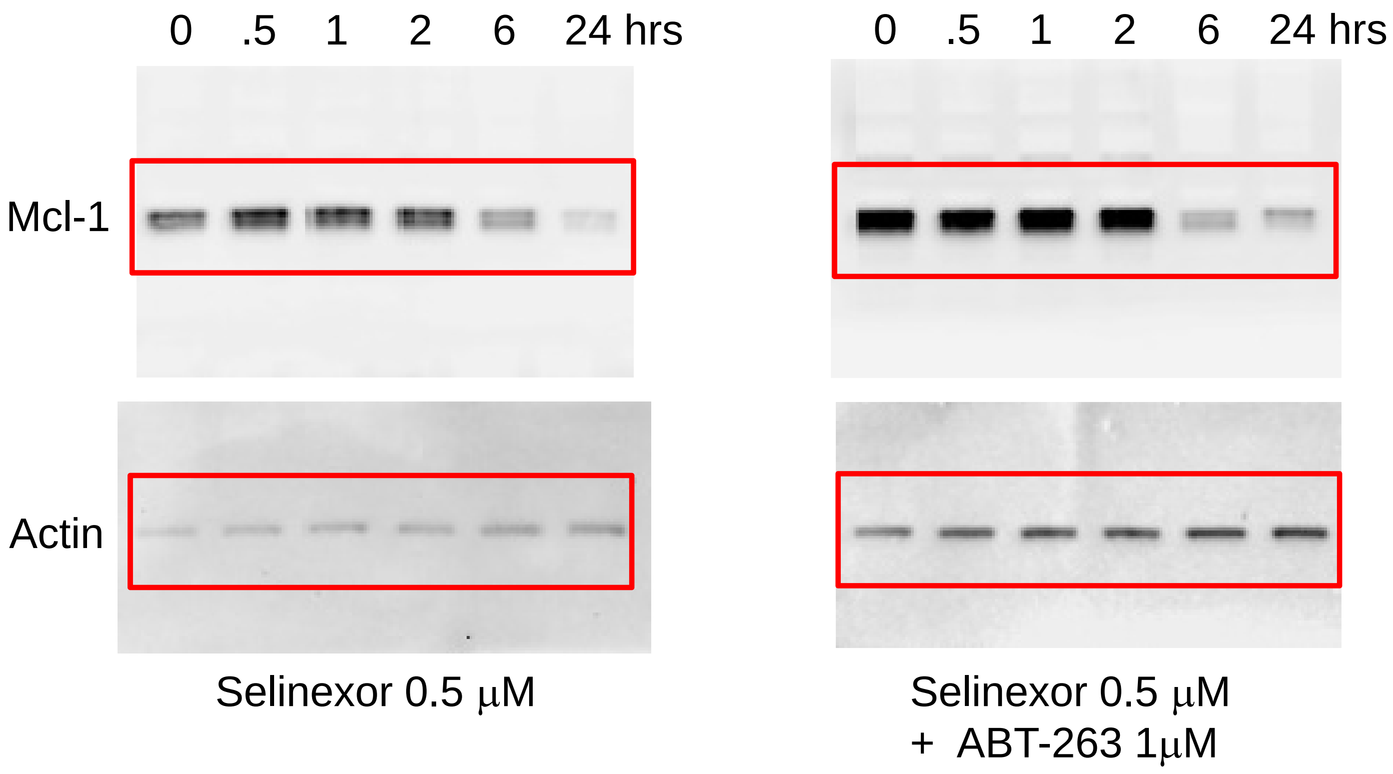
0 .5 1 2 6 24 hrs
0 .5 1 2 6 24 hrs
Mcl-1
Actin
Selinexor 0.5 μM
Selinexor 0.5 μM
+ ABT-263 1μM

Figure 5C_S

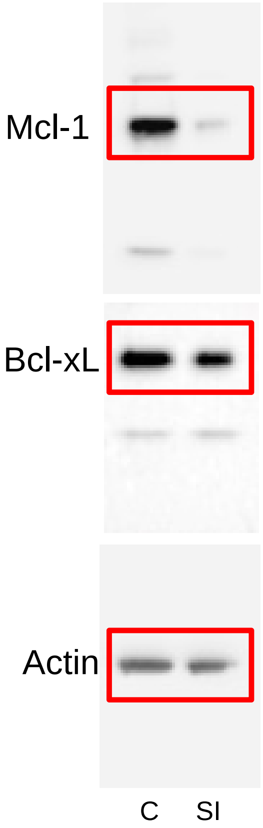

Supplement: Supplementary file 1 — Supplementary Dataset 1 [file 41598_2018_33784_MOESM1_ESM.pdf]
